# “A palliative end-stage COPD patient does not exist”: a qualitative study of barriers to and facilitators for early integration of palliative home care for end-stage COPD

**DOI:** 10.1038/s41533-018-0091-9

**Published:** 2018-06-20

**Authors:** Charlotte Scheerens, Luc Deliens, Simon Van Belle, Guy Joos, Peter Pype, Kenneth Chambaere

**Affiliations:** 1 End-of-Life Care Research Group, Ghent University & Vrije Universiteit Brussel (VUB), Ghent, Belgium; 2 Department of Internal Medicine, Ghent University, Ghent, Belgium; 3 Department of Family Medicine and Primary Health Care, Ghent University, Ghent, Belgium; 4 Department of Medical Oncology, Ghent University Hospital, Ghent, Belgium; 5 Department of Respiratory Medicine, Ghent University Hospital, Ghent, Belgium

## Abstract

Early integration of palliative home care (PHC) might positively affect people with chronic obstructive pulmonary disease (COPD). However, PHC as a holistic approach is not well integrated in clinical practice at the end-stage COPD. General practitioners (GPs) and community nurses (CNs) are highly involved in primary and home care and could provide valuable perspectives about barriers to and facilitators for early integrated PHC in end-stage COPD. Three focus groups were organised with GPs (*n* = 28) and four with CNs (*n* = 28), transcribed verbatim and comparatively analysed. Barriers were related to the unpredictability of COPD, a lack of disease insight and resistance towards care of the patient, lack of cooperation and experience with PHC for professional caregivers, lack of education about early integrated PHC, insufficient continuity of care from hospital to home, and lack of communication about PHC between professional caregivers and with end-stage COPD patients. Facilitators were the use of trigger moments for early integrating PHC, such as after a hospital admission or when an end-stage COPD patient becomes oxygen-dependent or housebound, positive attitudes towards PHC in informal caregivers, more focus on early integration of PHC in professional caregivers’ education, implementing advance care planning in healthcare and PHC systems, and enhancing communication about care and PHC. The results provide insights for clinical practice and the development of key components for successful practice in a phase 0–2 Early Integration of PHC for end-stage COPD (EPIC) trial, such as improving care integration, patients’ disease insight and training PHC nurses in care for end-stage COPD.

## Chronic lung disease: Improving palliative care

Research in Belgium provides insights into the possible implementation of early palliative care (PC) for patients with chronic obstructive pulmonary disease (COPD). While many sufferers of end-stage COPD would benefit emotionally and physically from palliative home care in their final months, many only receive PC in their last few days. Charlotte Scheerens at End-of-life Care Research Group, Ghent University and Vrije Universiteit Brussel (VUB) and co-workers at Ghent University and Vrije Universiteit Brussel (VUB) conducted focus group discussions with 28 general practitioners and 28 community nurses to establish the main barriers to and facilitators for palliative home care implementation for end-stage COPD. Barriers included the unpredictability of the disease, lack of patient understanding of COPD, and complex communication issues between staff across primary care, hospitals and community settings. Identifying ‘triggers’ to implement PC—oxygen dependence, for example—and improving communication and understanding of COPD at all levels could facilitate early integration of palliative home care (PHC).

## Background

Chronic obstructive pulmonary disease (COPD) is one of the leading causes of death,^1^ with an illness trajectory characterised by a progressive and inexorable decline interlaced with acute exacerbations.^2^ People with end-stage COPD (which we chose to define as ‘mostly GOLD stage III or IV^3^ and low to very low functioning’, although no clear definition of severe, very severe or end-stage COPD is available in literature), mainly suffer from symptoms such as dyspnea, pain, fatigue, anxiety and low mood, leading to a poor quality of life in the final stages of the disease.^4^ Despite numerous therapies to treat symptoms, end-stage COPD impacts heavily on emotional and social functioning and daily activities.^4^ Their physical and psychosocial symptoms are poorly addressed.^5^

We know from former research that palliative care (PC), if integrated earlier than the final weeks of life with standard care, can offer support for these symptoms as they in fact signal PC needs, and may have a positive impact on people with end-stage COPD.^6,7^ The Global initiative for chronic Obstructive Lung Disease (GOLD) also recommends early intergrated PC as a way to improve symptoms that reflect PC needs (such as dyspnea, anxiety, pain, and fatigue), which would potentially be better treated if PC was not only introduced in end-of-life situations.^3^

However, there is no unifying definition or common understanding in literature of early integrated PC. This might be due to the polymorphous nature of integrated care itself.^8^ For this study, the meaning of early integrated PC can be derived from combining definitions of PC and Integrated Health Services given by the World Health Organization (WHO). The WHO definition of PC incorporates: (1) encouraged collaboration between all professional caregivers (which we define as general practitioners, specialist physicians, nurses, physiotherapists, dentists, pharmacists, midwives, and paramedics), in order to connect expertize; and (2) early assessment of PC, by integrating PC with disease based “curative” therapies.^9^ Furthermore, the WHO definition of Integrated Health Services emphasises on “the management and delivery of health services so that patients receive a continuum of preventive and curative services, according to their needs over time and across different levels of the health system.”^10^

Moreover, patients with end-stage COPD often die in intensive care units in hospitals or nursing homes rather than at home,^11^ whereas end-stage COPD patients actually prefer home care,^12^ and patients with all kinds of diseases wish to die at home.^13^ If PC is provided at home by a PC nurse or PC professional, it can also improve quality of life and care,^14,15^ increase the chance of dying at home^16^ and reduce the burden of symptoms.^17^ Moreover, early integrated palliative home care (PHC) can help to avoid hospital admissions and escalation of costs related to the final months of life for people with end-stage diseases.^18^ Lastly, a qualitative study has shown that end-stage COPD patients have indicated needs for PHC and fully accept early integrated PHC.^19^

Early integrated PHC for end-stage COPD patients in practice, however, is not without its challenges as the unpredictable illness trajectory and chance of survival can interfere with its early integration.^6^

In Belgium, COPD accounted for 10.7% of all deaths, which made it the third most common cause of death in 2015.^20^ Furthermore, a study in Flanders revealed that of all deaths from end-stage COPD in 2013, only 37.3% were referred to PC, of which 7.2% to PHC. For half of the referred patients, time of onset of PC was only six days prior to death.^21^ Reasons for not referring end-stage COPD patients were according to the physicians due to a lack of time, because PC was not meaningful, or that PC needs were addressed in standard care.^21^ As research on implementing early integration of PHC for end-stage COPD is thin on the ground, with studies only exploring patients’ acceptance of integrated PHC,^19^ investigating PC and end-of-life discussions for COPD in general^22^ or not specifically focusing on end-stage COPD,^16,23^ a more detailed examination of challenges and possibilities for early integration of PHC for end-stage COPD is needed to gain insight into reasons why early integration of PHC for end-stage COPD is currently lacking and how to tackle this.

Likewise, little is known about the opinions of involved parties in early integrating PHC for end-stage COPD, with the exception of patients' perspectives^19^ as well as those of pulmonologists.^24^ However, the views of general practitioners (GPs) and community nurses (CNs) on early integrated PHC for end-stage COPD are lacking. These perspectives are crucial in identifying specific reasons why implementing this type of care is difficult in end-stage COPD^25,26^ as GPs and CNs in Belgium are active in primary and home care and well informed about PHC services. In order to gain useful data for clinical and policy-related solutions, this study aims to identify (1) barriers and (2) facilitators from the perspective of GPs and CNs for early integration of PHC in standard care for people with end-stage COPD. This qualitative study is performed as a part of a larger study to develop a complex phase 0–2 intervention trial on early integrated PHC for end-stage COPD (EPIC) in Flanders, Belgium.

## Results

### Participant characteristics (Table 1)

Three focus group interviews with GPs and four with CNs were held with a total of 28 GPs (*n* = 8, *n* = 10, *n* = 10) and 28 CNs (*n* = 4, *n* = 7, *n* = 5, *n* = 12) that attended one of seven focus groups. The majority of participants were between 40 and 60 years old, and 32 were male. Clinical working experience was variable, with the largest groups (each seventeen participants) working for zero to nine years and working for 20–29 years. 32 of 56 participants did not introduce PC to end-stage COPD patients in the past year.

**Table 1:** Characteristics of participating general practitioners and community nurses (*n* = 56)

	General practitioners			Community nurses				Total
Characteristics	FG1 (*n* = 8)	FG2 (*n* = 10)	FG3 (*n* = 10)	FG4 (*n* = 4)	FG5 (*n* = 7)	FG6 (*n* = 5)	FG7 (*n* = 12)	56
*Sex*								
Male	4	4	4	2	3	4	3	24
Female	4	6	6	2	4	1	9	32
*Age*								
≤29	1	1			3		2	7
30–39	1	2	2		2		2	9
40–49	1	4	1	4	1	1	4	16
50–59	4	1	1		1	4	4	15
60–69	1	2	6					9
≥70								
*Practice location*								
Urban	8		10	4		2		24
Semi-urban or rural		10			7	3	12	32
*Number of end-stage COPD patients cared for in the last year*	(1 non-response)	(1 non-response)						
None			4	1		1	3	9
1–9			1	3	1	2	9	16
10–19	4	1	1		5	2		13
20–29	1	1	4		1			7
≥30	2	7						9
*Number of end-stage COPD patients introduced to palliative care in the last year*								
None	5	2	7	1	4	2	11	32
1–3	2	8	3	3	2			18
4–6					1	3	1	5
7–9								
≥9	1							1
*Active in a palliative home care team*								
Yes		4^a^	3^a^	2^b^	1^b^			10
No	8	6	7	2	6	5	12	46
*Clinical work experience (years)*								
0–4	1	2	1		4		3	11
5–9	1	1	0		1		3	6
10–19	1	2	2	2		1	3	11
20–29	3	3	2	2	2	2	3	17
≥30	2	2	5			2		11

^a^General practitioners were recruited as members of a LOK group. Without our prior knowledge, we found out they were part of a palliative home care team as palliative care physicians

^b^Community nurses were recruited solely because of their experience as a community nurse. Without our prior knowledge, some of them have had experience as a palliative home care nurse in the past or as a second job. One participant was recruited in FG4 because another participant canceled. This participant was a palliative home care nurse in the past, but is currently a full-time researcher on palliative care

As we recruited GPs through local peer review groups and CNs through area-specific group meetings for six of the seven focus group conversations, not all participants of these focus groups met the predefined inclusion criteria: 11 of 56 participants did not have five years or more clinical working experience, and nine participants did not care for at least three end-stage COPD patients in the last year. We also found out during the focus groups that ten participants were member of a PHC team as a PHC physician or PHC nurse, either currently or in the past, without our prior knowledge.

### Barriers to early integration of PHC for end-stage COPD (Table 2)

#### Disease trajectory of end-stage COPD

Because of the unpredictable disease trajectory of end-stage COPD (1.a), people with end-stage COPD often experience unexpected exacerbations or other infections, and a sudden death. This made it difficult to decide when or whether PHC is needed. According to participants in FG2gp and FG5cn, it was also unclear when to go from curative care to PHC as the deteriorating functioning of the patient is often invisible (1.b) to the professional caregiver as the disease evolves slowly.

**Table 2:** Barriers according to general practitioners (GPs) (FG1_gp_, FG2_gp_, and FG3_gp_) and community nurses (CNs) (FG4_cn_, FG5_cn_, FG6_cn_, and FG7_cn_) for early integrating palliative home care in standard care for patients with end-stage COPD

1	Disease trajectory of COPD	1.a: Unpredictable exacerbations and death (FG1gp, FG2gp, FG3gp,FG5cn, FG7cn) 1.b: Invisible deterioration of functioning (FG2gp, FG5cn)
2	Perceived patient attitudes	2.a: Lack of disease insight: 1. Not understanding the severity of the disease or realizing the possibility of death (FG1gp, FG2gp, FG3gp, FG5cn, FG7cn) 2. Denial of the severity of the disease (FG2gp, FG5cn) 2.b: Resistance to care 1. The wish to be left on their own (FG2gp, FG5cn, FG6cn, FG7cn) 2. The wish to lead the life as they wished, accepting the consequences (FG1gp, FG3gp, FG5cn, FG7cn) 2.c: Resistance towards palliative (home) care because of the association with death (FG2gp, FG3gp, FG6cn, FG7cn)
3	Professional caregiver practices	3.a: Lack of a coherent and proactive care plan 1. No cooperation between professional caregivers involved in home care (FG1gp, FG4cn, and FG7cn) 2. Conflicting therapy and treatment between professional caregivers (FG3gp, FG5cn) 3.b: Insufficient experience with and negative vision of palliative home care for end-stage COPD 1. No experience in clinical practice with palliative (home) care for end-stage COPD (FG1gp, FG2gp, FG3gp, FG5cn, and FG7cn) 2. Professional caregivers continue to give life-prolonging care as added value of palliative (home) care for people with end-stage COPD is not clear (FG1gp, FG5cn)
4	Education for professional caregivers	Not enough focus on knowledge and advantages of palliative (home) care for end-stage COPD in professional caregivers’ basic and continuing education (FG2gp, FG3gp, FG5cn, and FG6cn)
5	Healthcare and palliative home care system characteristics	5.a: Consultations: not enough time during consultations to start talking about palliative home care and further care (FG4cn) 5.b: Coordination between hospital and home care 1. Lack of guidance on how to early integrate palliative home care to allow the patient to stay and die at home (FG1gp, FG2gp, and FG6cn) 2. Discharge from hospital to home situation without concrete guidelines (FG1gp, FG3gp) 5.c: Reimbursement system for palliative home care services 1. Palliative status for palliative home care is based on predictability of death (FG2gp, FG5cn) 2. Palliative reimbursement of palliative home care is restricted to 3 months (FG2gp, FG3gp, and FG7cn)
6	Communication	6.a: Inter-professional communication 1. Not knowing each other well enough for proper communication (FG2gp, FG3gp, FG5cn, and FG6cn) 2. Unclear who takes initiative to introduce palliative home care to end-stage COPD patients (FG3gp) 3. Not understanding each others' messages (FG2gp) 6.b: Communication between caregiver and end-stage COPD patient 1. Not discussing palliative care (needs) in detail during consultations with end-stage COPD patients (FG2gp) 2. Difficulties for professional caregivers to talk about palliative care needs with their end-stage COPD patients (FG2gp, FG3gp, and FG4cn) 3. Patient–family relationship can prevent communication on palliative home care (FG1gp, FG5cn, and FG7cn) 4. Professional caregivers fear talking about palliative home care because of the patient’s reaction (FG5cn)

I once saw a terminal COPD patient, with heavy exacerbations, as if he was almost gone, but he can now live further and wrestle through all of that again. And I think that maybe that has something to do with it, that we [professional caregivers] don’t quite see it [deterioration] like that, right? (FG1, GP).

#### Perceived patient attitudes

A lack of disease-insight (2.a) was mentioned, as some end-stage COPD patients did not seem to understand cognitively the severity of end-stage COPD and the possibility of death. This made it difficult for professional caregivers to start talking about PHC because the patient did not grasp the need for it. Participants associated this attitude more with their end-stage COPD patients than patients with other diseases such as cancer. Denial of the severity of end-stage COPD even when aware of the possible negative consequences was another example of lacking disease insight:

You also have these [end-stage COPD] patients, we see that visually, whose health is declining. Blue lips, blue as… They rarely accept that when you tell them [that they are going to die] - No, no… I am not going to die. That is the denial, that is that denial (FG5, CN).

Resistance towards care (2.b) was also mentioned, an attitude which depended on the patient’s personal context and personality. For example, some patients did not want further help from professional caregivers because they wanted to be left alone, while others refused it because of the wish to live life the way they wanted, thereby accepting the consequences. A participant explained that an end-stage COPD patient kept on smoking even when severely ill, stating it was too late for help anyhow. Other patients seemed to wait too long to contact a doctor, which made early integration of PHC impossible as they died before care could be given.

But, information… there are many who do not want to hear it [information about further care possibilities such as palliative home care], they [the patient] tell us [professional caregivers] to leave them alone (FG7, CN).

Finally, participants mentioned that the attitude towards PHC was one of resistance because of the perceived affiliation with death (2.c), as seen in this quotation:

We [professional caregivers] try to stimulate that [palliative home care] for our [end-stage COPD] patients, but it is really hard. Palliative care has a bad connotation, you know. When patients hear they are palliative, they believe they are going to die (FG5, CN).

#### Professional caregiver practices

The lack of a coherent and proactive care plan (3.a) in professional caregiver practices formed a barrier, firstly because professional caregivers experienced care coordination problems in the home situation of patients with end-stage COPD. For example:

On improving care: you have the cleaning help, the family help, the nurses and so on, and they all have something to say about the [end-stage COPD] patient, like maybe you should try this or that sometime, maybe try that again, and then you, the general practitioner, arrives there, and there you are, with your scientific background and all the scientific evidence that you have learned, and all of those suggestions are fired at and you have to say “yeah, but that will not help, and that will not help either, and I sometimes find it difficult, that everyone [professional caregivers] has their opinion (FG1, GP).

Secondly, conflicting therapies between professional caregivers were said to prevent the early integration of PHC as well:

To me, a good general practitioner is someone who does nothing. He only manages and says “I think you are suffering from that illness, you should go see that specialist physician.” I think that is great. Because they cannot know everything, I fully agree. But, too often, you see general practitioners who think they have the answer, while they are totally wrong and that gives complications when it comes to patient compliance. Like when you [and end-stage COPD patient] show up with a specialist’s advice and your general practitioner says “hmm, you should not do that”. Come on, that cannot happen (FG5, CN).

Next, insufficient experience with and a negative vision of PHC for end-stage COPD (3.b) was noted during the focus groups as PHC in itself was either not well known or its usefulness for end-stage COPD was not clear to participants due to a lack of experience in PC for this particular patient group. During the focus groups, participants often claimed that ‘a palliative end-stage COPD patient does not exist’. Others asked the moderator to explain what PHC could do for people with end-stage COPD:

The reason I would not immediately use PHC is that I need to know what they can offer in that context. So we want them to be able to offer comfort at a critical moment. But what can they do for someone who is suffocating? So then we need to hospitalize them after all (FG2, GP).

Related to this, professional caregivers did not clearly see the added value of early integrated PHC for end-stage COPD as PC is perceived to curtail all curative options for the patient. Stopping curative care and starting PHC was said to feel unnatural, especially for GPs, as they want to cure the patient.

#### Education for professional caregivers

Basic and continuing education about PHC and its advantages for end-stage COPD seemed to be lacking, which also influenced the barriers about professional caregiver practices:

I wonder, if we, as general practitioners, would be better educated and could prescribe oxygen, how we could quickly move on to be giving oxygen. I think that would prevent a lot of hospitalizations (FG3, GP).

#### Healthcare and PHC system characteristics

Timeslots for professional caregivers’ consultations which are too short (5.a), due to the fact that professional caregivers are paid per consultation, prevented discussions about early integrated P(H)C as this topic requires a lot of time to explain properly. Furthermore, coordination between hospital and home care (5.b) was inefficient, observed in a lack of guidance on how to early integrate PHC into the home situation of an end-stage COPD patient in order to keep the patient at home until death. Also, a lack of concrete guidance after discharge from hospital to home was mentioned, with end-stage COPD patients sometimes leaving the hospital without knowing what the next steps of care are:

I [general practitioner] never knew anything [of information given by someone] from the hospital for COPD (FG2, GP).

Simply said: ‘go home and handle it [the situation where the end-stage COPD patient is in] yourself (FG2, GP).

The reimbursement system for PHC (5.c) in Flanders is by law restricted to three months, with the possibility of making a second claim,^27^ which can be interpreted as an existing structural barrier for referring patients to PHC. To receive this reimbursement, a patient needs to have a legal palliative status, which depends on life expectancy, i.e., between three months and 24 h before death (this rule was still legal at the time of the focus groups). As the unpredictable disease trajectory of end-stage COPD makes it hard to predict whether a patient is in the final three months of life, this was also for the participants seen as a barrier. Although early integrated PHC can be provided if patients do not have this status, and costs related to PHC are reimbursed even if patients are still alive after three months, GPs and CNs saw this restriction of three months as a psychological obstacle to early integrating PHC:

Three months, right, if you want to request palliative care for three or six months, we do not know whether that will be the case [for a end-stage COPD patient], and that keeps you from proposing this [palliative home care] to the patient, because of that palliative status (FG3, GP).

#### Communication

*A* lack of proper communication between the involved professional caregivers (6.a GP), pulmonologist, CNs and PHC nurses) was observed due to different roles and perspectives on care:

Specialists also speak from an ivory tower. I’m thinking of a woman [with end-stage COPD] now, who is terminal, and sure, she has a lot of pain and she uses tramadol [an opioid]. Step one in the treatment, according to them [pulmonologists], is medication because it suppresses the respiratory system. But, come on. That is easy to say behind your little desk, wearing your suit, is it not? (FG2, GP)

There was also confusion about who should take the initiative to early integrate PHC for the patient, along with miscommunications in referral letters, cited by this quote:

Referral letters mentioning no possibilities for curative treatment for a cancer patient [from oncologists to general practitioners] often state: “referral to palliative team”. But with terminal COPD it [the referral letter from pulmonologists] just says “lung function borders livability”. (FG2, GP).

Another barrier was communication between professional caregivers and patients with end-stage COPD (6.b), as talking about further care and PHC needs with the patient was mentioned to be difficult during consultations, especially when patients’ family members were involved.

If his wife is not at home, then he [end-stage COPD patient] is incredibly chatty and he can pour out his heart: “and I do not want to live anymore and I want to die.” And when his wife gets back, the first thing he says: “do not say anything, my wife is here”. But come on, we only get to talk to him for fifteen minutes and the rest of the day he is with her. You realize he cannot discuss his illness with her, right? (FG5, CN).

Lastly, all focus groups mentioned communication problems due to the terminology of PHC, stating that the term has negative connotations for end-stage COPD patients and professional caregivers, as it implies impending death.

### Facilitators for early integration of PHC for end-stage COPD (Table 3)

#### Trigger moments

Participants expressed the need for trigger moments in the course of the disease trajectory of end-stage COPD as a way to facilitate early integration of PHC. Examples were after a hospital admission (1.a) as a moment to start talking about the future or reorganising care, a couple of exacerbations (1.b), when the patient becomes oxygen-dependent (1.c) or housebound due to a loss of functioning (1.d). These moments were seen as turning points when the patient realises the severity of the disease more clearly:

**Table 3:** Facilitators according to general practitioners (GPs) (FG1_gp_, FG2_gp_, and FG3_gp_) and community nurses (CNs) (FG4_cn_, FG5_cn_, FG6_cn_, and FG7_cn_) for early integrating palliative home care in standard care for patients with end-stage COPD

1	Trigger moments	1.a: Hospital admission 1. After hospital admission, a moment to start talking about the future (FG1gp, FG2gp, FG3gp, FG4cn, FG6cn) 2. After hospital admission, a moment to reorganize care (FG2gp) 1.b After a couple of exacerbations (FG2gp) 1.c: When an end-stage COPD patient becomes oxygen-dependent (FG2gp, FG3gp) 1.d: When an end-stage COPD patient is confronted with loss of functioning and becomes housebound (FG1 gp, FG2gp, FG5cn)
2	Involvement of informal caregivers	Increase knowledge about advantages of palliative home care for informal caregivers from patients with end-stage COPD (FG1gp, FG2gp, FG3gp, FG5cn)
3	Education for professional caregivers	More focus on early integrated palliative home care for end-stage COPD and concrete implementation in clinical practice in education for professional caregivers (FG5cn)
4	Healthcare and palliative home care system characteristics	Start advance care planning as a standard procedure for end-stage COPD patients living at home (FG1gp, FG2gp, FG3gp, FG4cn, FG7cn)
5	Communication	5.a: Communication between professional caregivers and end-stage COPD patients 1. Talking about practical matters can help professional caregivers to start talking about palliative home care (FG2gp, FG3gp) 2. Inform end-stage COPD patients clearly and firmly about their disease and future (FG4cn) 3. Better explanation of the term early integrated palliative home care can help acceptance for end-stage COPD patients: talk about it as comfort care, psychosocial support (FG2gp) 5.b: Communication between professional caregivers: appoint a care coordinator who facilitates the care transition to early integrated palliative home care (FG3gp, FG5cn, FG6cn)

Someone [end-stage COPD] who goes home after hospital and gets oxygen, that is an important thing to work on as a team [of professional caregivers]. And that [early integrated PHC] is something we [professional caregivers] could then discuss (FG2, GP).

For example, I think that starting oxygen at home is quite the occasion [for early integrating PHC]. After all, it announces a huge phase (FG2, GP).

#### Involvement of informal caregivers

Mainly GPs thought that providing more information about PHC and increasing positive attitudes towards it among informal caregivers (such as family members, volunteers of PHC teams) could encourage the latter to support the end-stage COPD patient with early integrated PHC:

We [general practitioners] are often asked by the family to come and talk without the patient being present. And then we discuss what will happen, what the palliative home care team could do, practical agreements (FG2, GP).

#### Education for professional caregivers

When talking about knowledge and care for end-stage COPD, there was an urgent need for more information about early integrated PHC for end-stage COPD and clinical implementation of it given in standard and further education of professional caregivers. This could better prepare professional caregivers in supporting end-stage COPD patients.

#### Healthcare and PHC system characteristics

Advance care planning (ACP) as a standard procedure in clinical practice for all end-stage COPD patients could facilitate conversations about the future, further wishes and needs. This could trigger professional caregivers and COPD after end-stage patients to think about integrating PHC earlier. ACP is already a practice in nursing homes in Belgium:

Because at that moment [going to a nursing home] there is a very important changeover in the life stage of a person. And because it is actually common to do advance care planning for someone who ends up in a nursing home. That is a procedure (FG2, GP).

Reply: we should do this for all our chronic ill patients (FG2, GP).

#### Communication

Enhancing communication from professional caregivers towards end-stage COPD patients (5.a) could be a facilitator, by using practical matters such as ‘where would you like to die’ as a way to start talking about early integrated PHC. Another possibility could be giving clear-cut information about end-stage COPD and future chances of survival in order to make the patient realise the severity of their disease:

You do not need that [advance directive] if you are already at the point of dying. But for the things that might come. I think about an end-stage COPD patient who always says that “they [professional caregivers] will never put me on those machines [in the hospital] anyway, right?”. [General practitioner says:]”Sure, but then we do really have to put that on paper, right?” And that is where you have a lead [to start talking about early integration of PHC]. Those practical questions are hints to talk about how far you want to go [in future care] (FG2, GP).

Patients should be correctly informed about further possibilities, about what medical care can still do for them. And then the conversation should mainly be about what the patient still wants and what he or she still expects and, and good agreements will have to be made about what will and what will no longer happen to the patient. And if hospitalization is out of the question, how are we [professional caregivers, patient and informal caregivers] going to organize the care package, and especially, with what objective? (FG4, GP).

Furthermore, even if some respondents felt the need to change the term PHC to supportive home care, others stated that focusing on symptom management, comfort and psychosocial support in conversations with end-stage COPD patients could also help the latter to accept the content of P(H)C:

You do not have to use the term palliative if you can say okay, from now on we will give you [end-stage COPD patient] maximum comfort and we will do everything to take care of you as good as possible without calling that directly palliative [care] (FG2, GP.)

Finally, improving communication between professional caregivers (5.b) by appointing a care coordinator to facilitate the information flow between professional caregivers involved in hospital and home settings and integrating different professional caregivers’ perspectives could increase early integration of PHC. If specialist physician’s medical information would be combined with information from home and primary caregivers this could provide a better view of the patient’s personal, medical and social context. A care coordinator could also introduce the advantages of early integrated PHC to the patient and their informal caregivers, as this function could have more time for these conversations than other currently involved professional caregivers:

Looking at each end-stage COPD patient to see which network can be provided and making connections with specialists' network. “Which nurse, which GP would you [the patient] like?” Then every end-stage COPD patient will have their own network up to informal care (FG5, CN).

## Discussion

### Main findings

The results of this study have revealed perceived barriers and facilitators from the perspective of general practitioners (GPs) and community nurses (CNs) to early integration of palliative home care (PHC) in standard care for people with end-stage COPD in Flanders, Belgium. The categories of barriers were (1) unpredictable exacerbations and death in COPD and invisible deterioration of functioning; (2) perceived patient attitudes such as a lack of disease insight and resistance towards care; (3) professional caregiver practices with a lack of a coherent and proactive plan, insufficient experience and a negative view of PHC for end-stage COPD; (4) not enough focus on knowledge and advantages of PHC and palliative care (PC) for end-stage COPD in professional caregivers’ basic and continuing education; (5) healthcare and PHC system characteristics: too short consultations, insufficient coordination between hospital and home care, and a reimbursement system for PHC that is based on life expectancy; and (6) communication: a lack of and unclear communication about care possibilities for end-stage COPD patients between professional caregivers, and a lack of clear information about PHC between professional caregivers and their patients.

The categories of facilitators were (1) trigger moments to start talking about early integration of PHC: such as after hospitalisation, after a couple of exacerbations, when an end-stage COPD patient becomes oxygen-dependent or becomes housebound; (2) involvement of informal caregivers in early integrated PHC for COPD; (3) information about the advantages of early integrated PHC for end-stage COPD in professional caregivers’ education; (4) including advance care planning (ACP) as a part of healthcare and PHC systems and (5) enhancing communication between professional caregivers by installing a care coordinator, and enhancing communication between professional caregivers and end-stage COPD patients by explaining better and giving practical examples of what early integrated PHC could mean for end-stage COPD.

### Interpretation of findings in relation to previous research

The following barriers were in line with previous research on (communication about) PC in general, early integration of PHC or PC for end-stage COPD: 1.a: unpredictable exacerbations and death;^6^ 3.b.2: continuation of life-prolonging care in end-stage COPD;^28^ 5.a: lack of time during consultations to start talking about PHC and further care;^28^ 5.b: no coordination between hospital and home care;^24^ 6.b.1: not discussing PHC, PC and PC needs in detail during consultations;^29^ 6.b.4: professional caregivers’ fear of talking about PHC because of the patient’s reaction.^30^

For facilitators we saw similarities with former studies on trigger moments 1.a: after hospital admission,^31^ and 1.c: when an end-stage COPD patient becomes oxygen-dependent;^32^ 3) professional caregivers’ education, with the importance of providing more focus on (implementation of) early integrated PHC;^24,33^ 4: health system and PHC system characteristics with reported advantages of ACP as a way to introduce PHC;^24^ 5.a.2: enhancing communication between professional caregivers and end-stage COPD patients by better informing the latter about PHC possibilities;^24^ 5.b: improving communication between professional caregivers by appointing a care coordinator.^24^

Due to the specific focus on early integrated PHC for end-stage COPD, this study also identified new insights into barriers on conflicting therapies and insufficient communication between professional caregivers and a lack of guidelines after hospital discharge. A common denominator might be insufficient or non-existent communication between hospital and home care settings.^24^ Professional caregivers active in hospital and home care might need to cooperate better and more often. By doing this, one could adjust care and therapies more adequately and better meet the patient’s wishes,^34^ while not forgetting to involve the patients and their informal caregivers in discussion about care. One option could be an electronic patient file accessible to the patients, their informal caregivers, and the professional caregivers in the hospital, the primary and the home care settings.^35^ This electronic patient file could contain a classification system that emphasises patients’ (PHC) needs and functioning instead of the disease, such as the comprehensive ICF core set for COPD, developed by the World Health Organization.^36^ Another possibility could be organizing multidisciplinary consultations consistently, each time a serious deterioration of functioning occurs, similar to multidisciplinary oncology consultations in Belgium. More research is needed to explore whether these examples could work for early integrated PHC in end-stage COPD.

Although it is known that PC and PHC increases quality of life for people with end-stage COPD when integrated early,^7^ the content of PHC needs adaptation if integrated before the terminal stage, depending on the disease population and the personal needs of the patient.^37^ Research has shown the need for management of troublesome symptoms and short-term PC if integrated early.^6^ Managing breathlessness or relieving psychosocial symptoms which are often seen in end-stage COPD despite receiving optimal medical care^5^ might require the involvement of other care professionals besides a PHC nurse, such as a physiotherapist, psychologist or social worker. Re-evaluating the content of PHC if given early and integrated for end-stage COPD is therefore necessary in order to be fully effective.

The large volume of results on professional caregiver-patient communication showed plenty of room for improvement. Participants claimed that some end-stage COPD patients did not fully understand the disease, sometimes refused care and often interpreted the term PC or PHC as a sign of impending death. However, previous research found that patients with end-stage COPD did express the desire to talk about end-of-life care^30^ and fully accepted PHC and early integrated PHC.^19^ This could thus mean that participants in our study either misinterpreted their end-stage COPD patients’ wishes and communication preferences about PHC, or that their end-stage COPD patients did not clearly share their care needs which would confirm other research finding that patients often do not fully understand the severity of end-stage COPD,^38^ or did not know what future care they would prefer.^22^ In contrast, another study found that patients did convey the need for involvement and education about end-stage COPD and PC, which could improve PC communication.^39^ A previous trial tested patient feedback by giving self-reported patient questionnaires on end-of-life preferences for communication, therapy and experiences. These were then given to the involved professional caregivers which resulted in better patient-professional caregiver communication.^40^ As literature and the results of our study did not provide a clear answer to these communication issues between professional caregivers and patients, further testing of communication systems is suggested, while improving undergraduate and postgraduate education for professional caregivers on bad news delivery, ACP and shared decision making.

The unpredictable disease trajectory of end-stage COPD was mentioned as a factor impeding (timely) referral to and conversations about early integrated PHC, somewhat confirmed by research stating the need for clear identification criteria for pulmonologists to introduce PC in a timely manner.^24^ The trigger points identified in the results of our study could respond to this need, as they signal an increase in PHC needs due to a decline in functioning of the end-stage COPD patient such as after a hospital admission, a couple of exacerbations, oxygen-dependency or becoming housebound. These trigger moments were not related to life expectancy as the latter was seen as an inappropriate basis for deciding whether early integrated PHC was needed, which is in line with a study proving that criteria to predict survival in end-stage COPD do not work.^41^ Moreover, at the time of the focus group conversations, eligibility for PHC in Belgium was dependent on a palliative status based on life expectancy (less than 3 months before death), which was seen as a psychological barrier to early integrating PHC as the unpredictability of COPD prevents professional caregivers from deciding whether an end-stage COPD-patient is likely to survive for three months. Nevertheless, somewhat contrary to our results, a previous qualitative study with end-stage COPD patients found that admission for exacerbation was considered too chaotic and not an appropriate occasion to discuss PC, although it could be a milestone leading to PC discussions.^31^ Pulmonologists also stated that conversations about treatment preferences should be initiated when an end-stage COPD patient is stable.^32^ It is important to mention that the trigger moments in the results of our study could give rise to an opportunity for talking about early integrated PHC as these moments could help the end-stage COPD patient realise the severity of the disease, but initiating the conversations should take place when the end-stage COPD patient is back in a stable context, preferably at home, after the events had occurred. More research is needed to explore the feasibility of addressing PHC needs following the different triggers.

### Strengths and limitations

The research team involved in data analysis were people with different backgrounds including psychology, sociology, general practice, primary care, PHC, pulmonology, and oncology. This enhanced the interpretation of the data due to the multitude of perspectives. Furthermore, to the extent of our knowledge, this is the first qualitative study reporting GPs’ and CNs’ insights into barriers to and facilitators for early integration of PHC for people with end-stage COPD. We obtained a varied sample of GPs and CNs with different backgrounds, care experience and perspectives on PHC. The high number (56) of participants in seven different focus groups also constituted a key strength of this study as it improved transferability of the findings beyond the context of the individual participants’ experiences.

However, it is worth noting that due to altering recruitment strategies at the start of the study not all participants reached the inclusion criteria we predefined for the study. Eleven out of 56 did have less than five years working experience, and nine of 56 participants did not have COPD patients in their practice in the past year. This could have influenced the results as professional caregivers with less working experience or less experience with COPD patients might have faced difficulties in answering questions about early integration of PHC for end-stage COPD, as they could have lacked the clinical experience to relate their answers with. Nevertheless, we believe that due to the setting of a focus group, where groups were formed with other participants having many experience, this limitation did not compose any substantial problems to the quality of the conversations and to the results. During the focus group conversations, the experienced professional caregivers inspired the less experienced participants to reflect critically on the questions asked by the moderator. The strength of the answers from focus group conversations also relied on the vivid discussion between the participants who challenged each other in giving answers to questions. Therefore, we did not exclude the less experienced participants from the analysis as their participation helped in obtaining the results.

Furthermore, 32 of 56 participants had never introduced end-stage COPD patients to PC or PHC in the past year. This might have affected the results due to a lack of experience with PC or PHC for end-stage COPD between the participants. However, this does not mean that the participants did not know what PC or PHC can do for patients, as they had have experience with PC, but mainly for cancer patients. Therefore, we believe that these participants were able enough to form an opinion on why they never or hardly introduce PHC for end-stage COPD patients compared to cancer patients and what could be done to alter this.

Another limitation of this study was the lack of insights from other professional caregivers involved in care for end-stage COPD patients, such as pulmonologists and physiotherapists. Neither did we consult patients with end-stage COPD or informal caregivers. However, gaining insight into early integrated PHC was the primary focus of the study and therefore we interviewed professional caregivers active in primary and home care. Notwithstanding these limitations, the results could provide valuable information on the development of feasible interventions, practical implementation and policy-related recommendations on early integrated PHC for end-stage COPD.

### Implications for policy and practice, and future research

Given the complexity of implementing early integrated PHC for end-stage COPD, we suggest a multilevel strategy approach in order to successfully change related policy and practice.^42^ The micro level could be adapted by increasing patients’ insight into (end-stage) COPD and early integrated PHC using government-funded campaigns about PC and PHC on national television which could raise awareness of PC and PHC among the general population. Meso-level changes could be on focusing professional caregivers’ basic and continuing education more on clinical PHC practice through obligatory internships in PC and PHC settings, enhancing knowledge about (end-stage) COPD, PC and PHC needs, advantages of early integrated PHC, and focusing on skills in communication and ACP.

Finally, macro change by adapting the healthcare and PHC system would be needed, for example by disconnecting eligibility for palliative status and reimbursement of PHC-related costs from life expectancy and instead linking it to lower functioning, PC and PHC needs in end-stage COPD.^27^ Although the Flemish government has decided to change this system, the law has not yet been changed. Additionally, incorporating ACP as a standard procedure within early integrated PHC to facilitate patient-professional caregiver communication, and appointing care coordinators as an additional role in existing care could provide continuous support for end-stage COPD patients over different care settings.^42^ However this would require an economic costs and benefits analysis.

As this study was performed to develop the phase 2 EPIC trial, the results suggested using a comprehensive PHC model in the intervention with inclusion criteria representing high PHC needs as a proxy to start early integrated PHC for end-stage COPD.^43^ Based on our results, these inclusion criteria representing high PHC needs could be GOLD III or IV combined with low functioning such as frequent hospitalisations for COPD, exacerbations due to COPD, becoming housebound or oxygen-dependent. Key components could cover several dimensions of appropriate PHC, from improving patient’s disease insight, to training the PHC team in knowledge and therapy for end-stage COPD, and integrating care by trying to improve cooperation and communication between involved professional caregivers. Previous interventions in early PC and PHC for end-stage COPD mainly focused on caring for one symptom, for example managing breathlessness^44^ or provided training about one care aspect, such as nutrition.^23^ Instead, we suggest using several components to provide a holistic PHC approach, in order to fully tackle the lack of early integrated PHC for people with end-stage COPD.

## Conclusion

Our study uncovered barriers in terms of the disease trajectory, patient attitudes, professional caregivers’ practices, the healthcare and PHC system and communication problems. Facilitators provided possibilities at many levels for a successful implementation of early integrated PHC in practice or development of early integrated PHC interventions for end-stage COPD. This requires a multilevel approach with the involvement of professional caregivers active in hospital and home settings, while not forgetting to actively include end-stage COPD patients and informal caregivers in the process.

## Methods

### Study design

A qualitative approach using focus groups was chosen for its group dynamic features that stimulate interaction between participants and allow the moderator to use more active interview techniques than with face-to-face interviews.^45^ This approach was supported by the methodological orientation of grounded theory,^45^ as we constructed new insights based on data obtained from the focus groups. The research protocol and topic guides were approved by the Ethics Committee of Ghent University Hospital (Reference: 2016/0171).

### Study setting

The study was based in workplace settings in urban and semi-urban regions in Flanders, Belgium, in 2016, as it was part of the development of a phase 2 intervention on early integration of PHC for end-stage COPD in Flanders, Belgium.

### Study population and sampling

The study population consisted of GPs and CNs involved in primary and home care settings. In selecting the participants, three criteria were stipulated as a guiding line for recruitment: (1) Dutch speaking; (2) at least five years experience as a GP or CN; (3) having cared for at least three end-stage COPD patients. We also took into account variation in semi-urban and urban areas. We then used ‘convenience sampling’. With this technique, the sample was composed of participants or groups who met the criteria and who were available or signed up first.^46^ However, individual recruitment of GPs and CNs for focus group participation was difficult and we had to change strategy. Only one focus group with CNs was composed by gathering independent CNs from the same region. This was done by contacting individual CNs from one urban area by phone through a contact lis fron the Flemish Professional Association for Independent Nurses which is available online: http://www.verplegingthuis.be/.

As a solution, we used beside the convenience sampling technique, a purposive sampling technique which allowed the research team to select participants based on the researchers’ judgment. We organized focus groups which consisted either of one regionally composed group of GPs or one regionally composed group of CNs. This type of recruitment could offer us a sample of GPs and CNs representing a wide range of experience related to the topic (maximum variation sampling), even if not all participants would meet the inclusion criteria. For GPs these were local peer review GP groups (LOK): geographically determined groups of GPs from both individual and group practices. They meet four times a year to share and critically evaluate their medical practice (peer review) and to improve their quality of care. For CNs these were regionally composed groups from the National Association of Catholic Flemish Nurses and Midwifes (NVKVV) who meet monthly to discuss their practice and share work-related experiences.^47^

### Recruitment strategy

The participants were initially identified by a member of the research team and other key contacts who were either a GP, CN, or policy member of organisations involved in community care. Further recruitment for GPs was undertaken by contacting several people responsible for local peer review (LOK) GP groups in Flanders in person, by phone or e-mail. Further recruitment of CNs were recruited by contacting the “Wit-Gele Kruis”, a Flemish organisation for CNs. This organisation was asked to help with the recruitment by forwarding the call for participants to its members. With the help of this organisation, we contacted those responsible for regionally composed groups of CNs by phone to organise the focus group when that group had a meeting. Potential groups of GPs or CNs were invited to take part in a focus group with e-mails containing information about the study and participation. Suitable dates and venues were arranged with the people in charge of the local peer review GP groups or regionally composed CN group, if all group members agreed to participate in the study.

### Data collection

**Fig. 1:**
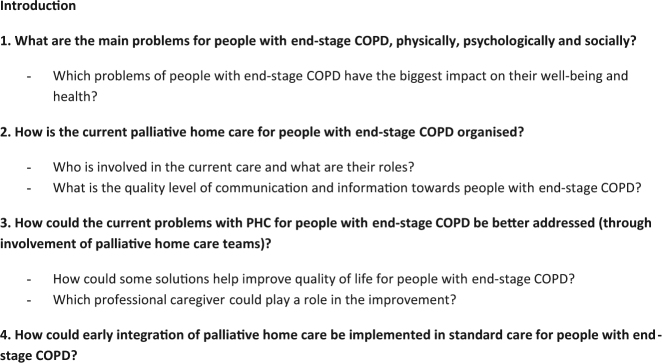
Topic list based on research questions

Recruitment and focus group conversations took place between March and September 2016. A semi-structured topic guide (pilot tested), consisting of four main questions and a set of prompts for each question, was developed and reviewed within a multidisciplinary research team of sociologists, a GP, a lung specialist and an oncologist (for content of the topic guide: Fig. 1). End-stage COPD was described during the focus group as mostly GOLD stage III or IV^3^ and low to very low functioning’.

Each focus group was moderated by one male senior researcher (sociologist or psychologist) and observed by one female junior researcher (sociologist or psychologist) who made field notes, all experienced in conducting focus groups due to training in former education and conducting qualitative research in previous studies. The focus groups took place in a quiet room, were conducted in Flemish, lasted on average one and a half hours and were audio taped, for which all participants gave informed written consent. All participants filled in a short questionnaire regarding their own demographic characteristics, clinical experience and experience with care and PC or PHC for end-stage COPD. After conducting two focus groups with GPs, the research team slightly adapted the topic list by leaving out the first question on perceived main problems for people with end-stage COPD, as this question did not lead to significant information regarding early integrated PHC. We continued recruitment and sampling until data saturation was achieved. Saturation was defined when no new themes on barriers and facilitators occurred during the focus group.

### Data analysis

The focus groups were completely transcribed verbatim. Then we used NVivo 9 software to code and analyse the data according to the research questions. Two researchers (CS and KC) first read and coded the data in themes which were derived from the data from four full focus group transcripts and compared similarities and differences in their analyses until a primary coding framework was constructed. Then all seven focus group transcripts were independently read, compared with the primary coding framework by the two researchers and these results were discussed with all members of the research team. Codes were added, modified or merged when necessary. A third and fourth researcher (PP and LD) made final changes to the codes, which were approved by the other two coding researchers. Once coding was finalized, all transcripts and the coding framework were revised and refined, resulting in (sub)categories of barriers and facilitators. Quotations were selected and approved by the research team to illustrate the results. Transcripts were not sent back to participants for correction but respondent validation of the results was undertaken by sending the results of the study by e-mail to all participating GPs and CNs for consent.

### Data availability

The data that support the findings of this study are available from the corresponding author (C.S) upon reasonable request.
